# LncRNA NEAT1 mediates progression of oral squamous cell carcinoma via VEGF-A and Notch signaling pathway

**DOI:** 10.1186/s12957-020-02028-x

**Published:** 2020-10-06

**Authors:** He Ke, Zhi-Bin Zhu, Rui Shu, Ai Hong

**Affiliations:** 1 Department of Stomatology, Chengdu Seventh People’s Hospital, Chengdu, Sichuan 610015 China; 2 Department of Orthodontics and Pediatric Dentistry, West China School of Stomatology State Key Laboratory of Oral Diseases, Sichuan University, Chengdu, 610041 China; 3 Department of Stomatology, The Third Affiliated Hospital of Sun Yat-sen University, Guangzhou, 510000 China

**Keywords:** lncRNA NEAT1, VEGF-A, EMT, Proliferation

## Abstract

**Background:**

lncRNAs and VEGF have been shown to have close connections with oral squamous cell carcinoma (OSCC). We explored the interaction between lncRNA NEAT1 and VEGF-A in OSCC.

**Methods:**

RT-qPCR was implemented to measure levels of lncRNA NEAT1 and VEGF-A in OSCC cell lines and normal cell lines. Cell functions then were checked after regulating the expressions of lncRNA NEAT1 and VEGF-A separately. Cell viabilities were examined with CCK-8 and apoptosis rate was checked with flow cytometry. Meanwhile, EMT-related genes E-cadherin, N-cadherin, Vimentin, and Snail and Notch signaling genes Notch1, Notch2, and Jagged were evaluated by RT-qPCR. IMR-1 was applied for impeding Notch signaling pathway. Later, cell viabilities, apoptosis, and EMT were assessed.

**Results:**

Expressions of lncRNA NEAT1 and VEGF-A were both increased significantly in OSCC cell lines especially in TSCC1 cell line. Suppression of lncNRA NEAT1 was associated with lower cell viabilities and EMT and higher apoptosis rate in the TSCC1 cell line. Meanwhile, knockdown of VEGF-A significantly repressed cell viabilities and EMT in the TSCC1 cell line. Magnifying functions of inhibited lncRNA NEAT1 Notch signaling pathway was obviously activated with overexpressions of lncRNA NEAT1 and VEGF-A. Adding IMR-1 significantly downregulated cell viabilities and EMT and sharply increased apoptosis in the context of lncRNA NEAT1 and VEGF-A overexpression.

**Conclusion:**

LncRNA NEAT1 may upregulate proliferation and EMT and repress apoptosis through activating VEGF-A and Notch signaling pathway in vitro, suggesting an underlying regulatory factor in OSCC. Nevertheless, further research is necessary to gain a greater understanding of lncRNA NEAT1 and connections with VEGF-A in vivo and in clinical study.

## Introduction

Oral squamous cell carcinoma (OSCC) is a head and neck malignant cancer with high aggressiveness that can invade adjacent bones, muscles, skin tissues, and local lymphoid tissues [1, 2], which takes up about 3% among all kinds of new cases of malignancies [3]. Though surgery and chemoradiotherapy techniques have made progress, the 5-year overall survival rate of OSCC is only 40–60% [4–6]. As for the pathogenesis of OSCC, smoking is considered as a primary factor and excessive alcohol drinking, betel nut chewing and human papillomavirus infection can also cause OSCC [7, 8]. According to previous studies, high proliferation and EMT are also urgent threats in treating OSCC [9, 10]. Although OSCC is the sixth most common cancer occurring in humans, its underlying mechanism is still unclear. Moreover, lack of treatment means leads to unfavorable prognosis of patients suffering from OSCC. Hence, it is necessary to dig out the nosogenesis of OSCC and mechanisms that can regulate progression of OSCC in order to reduce its morbidity and increase cure and survival rate of patients.

Occurrence and development of OSCC is a complicated genetic process. Previous research has often focused on protein-coding genes. In recent years, the role of long noncoding RNA has been explored, revealing that lncRNAs function in OSCC. For example, LINC01116 is high expressed in OSCC and suppression of LINC01116 in OSCC cells can upregulate miR-136 and inhibit FN1, resulting in deterring the progression of OSCC [11]. LncRNA AC007271.3 is upregulated in OSCC tissues and cell lines and overexpression promotes proliferation, migration, and invasion of OSCC cells [12].

Nuclear paraspeckle assembly transcript 1 (NEAT1), as a long non-coding RNA, is transcribed by familial tumor syndrome multiple endocrine neoplasia type 1 locus, which is an essential nuclear composition [13]. Moreover, its abnormal expression has been reported in many kinds of cancers including colorectal cancer, esophageal cancer, and laryngeal cancer [14–16]. In OSCC, NEAT1 was found to be highly expressed in saliva of OSCC patients and it is relatively low expressed in oral mucosa in normal people [17, 18]. Similar to LINC01116 and AC007271.3, LncRNA NEAT1 was also found to accelerate proliferation, migration, and invasion via sponging miR-365 in OSCC [19]. Nevertheless, research on the correlation between LncRNA NEAT1 and OSCC is still rare. Based on previous research, VEGF-A is an important factor in progression of OSCC. The expression of VEGF-A is regulated by lncRNA NEAT1 in colorectal cancer [20, 21]. Therefore, we hypothesized that there might be connections between LncRNA NEAT1 and VEGF-A in OSCC too.

Furthermore, the Notch signaling pathway is widely known as a promoter in progression of OSCC [22]. In epilepsy, it has been revealed that lncRNA NEAT1 could positively regulate Notch signaling pathway [23]. However, there has been no research to connect NEAT1 to NOTCH signaling in OSCC yet. For this reason, we supposed that NEAT1 might regulate OSCC progression through the Notch signaling pathway. Hence, in this study, we not only explored the role of NEAT in modulating the cellular functions in OSCC, but also aimed to unveil its correlation with NOTCH signaling with involvement of IMR-1, a Notch inhibitor [24].

## Materials and methods

### Cell culture

The human oral epithelial cell line HOEC and human OSCC cell lines (SCC-25, TSCC1, and CAL-27) were all acquired from American Type Culture Collection (ATCC, USA). SCC-25 and CAL-27 cell lines are human-derived tongue squamous cells. TSCC1 is oral squamous cell carcinoma cell line derived from human with OSCC. All cell lines were cultured in RPMI-1640 medium (Gibco™, USA) containing 10% fetal bovine serum (FBS, Gibco™, USA) at 37 °C, 5% CO_2_. After cell confluence reached 80%, 0.25% trypsin was used to digest cells for passaging. Cells were grouped as normal HOEC group and OSCC cell groups. Finally, cells in log phase were chosen for following experiments.

### Cell transfection

Two specific siRNAs of lncRNA NEAT1 and siRNA of VEGF-A were designed and offered by GenePharma (Shanghai, China). Meanwhile, overexpressed lncRNA NEAT1 and VEGF-A were produced with pcDNA3.1 (Invitrogen™, USA) and the oeNC was pcDNA 3.1 vector with inserted scrambled sequences. Based on different sequences, siRNAs were named siNEAT1-1, siNEAT1-2, and siNC. As for transfection, TSCC1 cells in log phase were resuspended and adjusted cell concentration to 10^5^ per well in 6-well plate. Then, cells were incubated until reached 50%. Next, serum-free medium was applied to replace medium used in incubation. Suppressions were divided into four groups, siNC, siNEAT1-1, siNEAT1-2, and siNEAT1-2 with siVEGF-A and overexpression plasmids were divided into three groups oeNC, oeNEAT1-2, and oeNEAT1-2 with oeVEGF-A. Afterwards, transfection contents were mixed with Lipofectamine™ RNAiMAX Transfection Reagent (Invitrogen™, USA) and cultured for 20 min at room temperature. Mixtures then were blended with cells in 6-well plate and cultured for 5-6 h at 37 °C, 5%CO_2_. Thereafter, cells were kept in culture for another 48 h in medium with FBS. RNA was extracted and then expression of RNA was checked with RT-qPCR.

### RT-qPCR

Total RNAs of cells in normal and tumor cell lines were extracted with TRIzol reagent (Invitrogen™, USA) following manufacturer’s instructions. After RNAs were collected, following specifications of High-Capacity cDNA Reverse Transcription Kit (Applied Biosystems™, USA), cDNAs were gathered through reverse transcription of RNAs. After that, PCR was performed to quantify expressions of RNAs through Applied Biosystems 7500 and 7500 Fast Real-Time PCR Systems (Thermo Fisher, USA). Specific primers were listed in Table 1. As for conditions of PCR, first was pre-denaturation, 95 °C, 5 min and following was denaturation, 95 °C, 30 s; then were annealing, 55 °C, 30 s and extension 72 °C, 30 s, 40 cycles. Expressions of target RNAs were calculated with 2^−△△Ct^ method [25].

**Table 1:** Sequences of primers

RNA	Sequences of primer
lncRNA NEAT1	forward 5′-ATGCCACAACGCAGATTGAT-3′ reverse, 5′-CGAGAAACGCACAAGAAGG-3 ′[26]
VEGF-A	forward 5′-AGGGTTTCGGGAACCAGAT-3′ reverse, 5′-CTGGCCTTGCACATTCCT-3 ′[27]
E-cadherin	forward 5′-TACACTGCCCAGGAGCCAGA-3′, reverse 5′-TGGCACCAGTGTCCGGATTA-3 ′[28]
N-cadherin	forward 5′-TCAGGCGTCTGTAGAGGCTT-3′, reverse 5′-ATGCACATCCTTCGATAAGACTG-3 ′[28]
Vimentin	forward 5′-GACGCCATCAACACCGAGTT-3′, reverse 5′-CTTTGTCGTTGGTTAGCTGGT-3 ′[28]
Snail	forward 5′-TCGGAAGCCTAACTACAGCGA-3′, reverse 5′-AGATGAGCATTGGCAGCGAG-3 ′[28]
Notch1	forward, 5′-ATGCAGAACAACAGGGAGGA-3′, reverse 5′-ACCAGGTTGTACTCGTCCAG-3′
Notch2	forward 5′-AATGGAGGCTATGGCTGTGT-3′, reverse 5′-GGCACATGCAAGAGAAGGAG-3′
Jagged1	forward, 5′-ACTTACCAGCCGTGTCTCAA-3′, reverse 5′-GCCTCGAGCACATTGACATT-3′
GAPDH	forward, 5′-GGTCTCCTCTGACTTCAACA-3′, reverse, 5′-GTGAGGGTCTCTCTCTTCCT-3 ′[26]

### CCK-8

After transfection was accomplished for 24 h, cells in log phase were harvested and seeded into 96-well plate with 5 × 10^3^ cells per well and each well contained 100 μL medium. In specific time points (24 h, 48 h, and 72 h), 10 μL CCK-8 were added and incubated with cells for another 2 h. Finally, optical density values (OD) of cells were measured at 450 nm wavelength with Multiskan™ FC microplate reader (Thermo Scientific™, USA). Abscissa was time and OD was the ordinate.

### Flow cytometry

Flow cytometry was performed to explore apoptosis of OSCC cells. After transfection for 48 h, cells in log phase were gathered and rinsed with cold PBS twice. Next, cells were suspended to adjust concentrations to 10^6^/ml. After that, cells were added into Annexin-V buffer (Beyotime, Shanghai, China) and mixed with 10 μL (20 μg/ml) Annexin-V-FITC and 5 μL (50 μg/ml) PI (Beyotime, Shanghai, China) for incubation at room temperature for 20 min without light. In the end, 400 μL Annexin-V buffer were appended and Attune NxT flow cytometer (Invitrogen, USA) was applied to analyze apoptosis rate of cells.

### Statistical analysis

All experiments were repeated three times independently and data were displayed as mean ± SD. All data were analyzed with SPSS 19.0 (IBM, USA) and GraphPad Prism 7 (GraphPad, USA). Results between two groups were measured with Student’s *t* test and one-way ANOVA was applied to check data among groups. *P* < 0.05 was considered to have statistical significance.

## Results

### LncRNA NEAT1 was highly expressed in OSCC cell lines and accelerated proliferation and EMT but suppressed apoptosis in OSCC cells

**Fig. 1:**
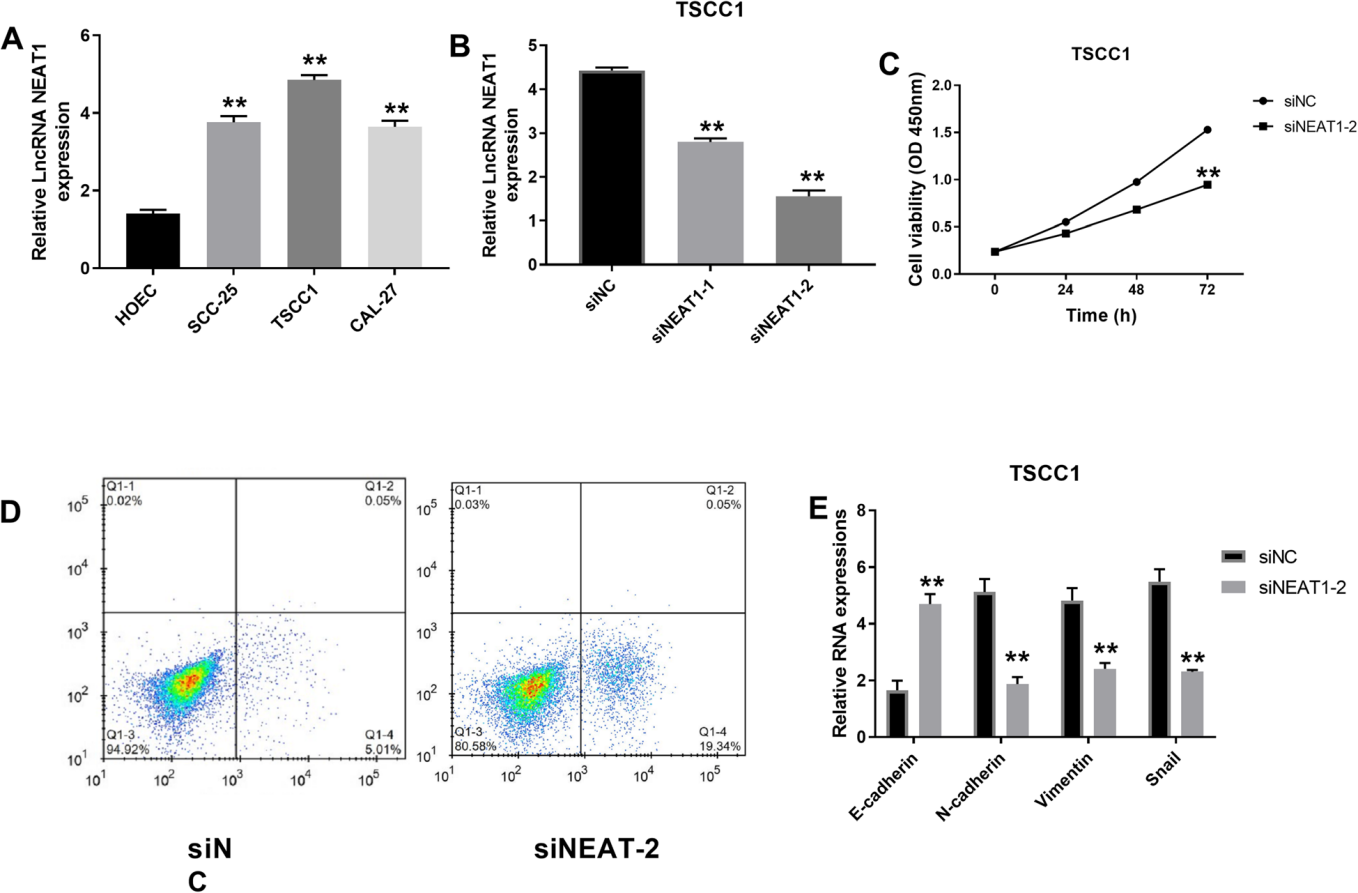
LncRNA NEAT1 highly expressed in OSCC cell lines and promoted proliferation and EMT but inhibited apoptosis. **a** Expressions of lncRNA NEAT1 in HOEC, SCC-25, TSCC1, and CAL-27 cell lines were examined with RT-qPCR with normalizing to GAPDH and the statistical test was comparing each of the OSCC cell lines to the HOEC cell line, **P < 0.05. **b** After suppression, levels of siNEAT1-1, siNEAT1-2, and siNC were checked by RT-qPCR with normalizing to GAPDH and the statistical test was comparing siNEAT1-1 and siNEAT1-2 to siNC, **P < 0.05. **c** CCK-8 was assessed to measure cell viabilities in TSCC1 cell line with siNC and siNEAT1-2 and the statistical test was comparing siNEAT1-2 to siNC, **P < 0.05. **d** Apoptosis rate of cells in TSCC1 cell line with transfected siNEAT1-2 and siNC were validated using flow cytometry and the statistical test was comparing siNEAT1-2 to siNC, **P < 0.05. **e** Expressions of E-cadherin, N-cadherin, Vimentin, and Snail were checked with RT-qPCR with normalizing to GAPDH and the statistical test was comparing E-cadherin, N-cadherin, Vimentin, and Snail in siNEAT1-2 group to E-cadherin, N-cadherin, Vimentin, and Snail in siNC group, **P < 0.05. Each experiment was repeated three times

According to results of RT-qPCR, expressions of lncRNA NEAT1 expressed significantly higher in OSCC cell lines than in normal HOEC cell line. Furthermore, among those OSCC cell lines, the TSCC1 cell line exhibited the highest level of lncRNA NEAT1 (Fig. 1a). For this reason, the TSCC1 cell line was selected for the following experiments. As lncRNA NEAT1 was highly expressed in OSCC cancer cells, two kinds of siRNAs were used to knock down lncRNA NEAT1 in cells. Based on RT-qPCR analysis, both silncRNA NEAT1-1 and silncRNA NEAT1-2 could notably downregulate lncRNA NEAT1 in TSCC1 cell line but silncRNA NEAT1-2 presented a more remarkable suppression of lncRNA NEAT1 in the cell line (Fig. 1b). Hence, silncRNA NEAT1-2 was chosen for the following experiments. After TSCC1 cells were regulated after transfection of silncRNA NEAT1-2 plasmid, cell viabilities were examined, revealing that silncRNA NEAT1-2 significantly decreased viabilities of cells compared to the negative control group (Fig. 1c). Meanwhile, results of flow cytometry showed that cells in silncRNA NEAt1-2 group acquired a higher apoptosis rate than cells with siNC group (Fig. 1d). Then, explored EMT-related factors were applied to check migration of TSCC1 cell line. Compared to siNC group, E-cadherin expression was obviously higher after lncRNA NEAT1-2 was inhibited while levels of N-cadherin, Vimentin, and Snail were remarkably lower (Fig. 1e).

### VEGF-A expressed higher in OSCC cell lines and promoted proliferation and EMT

**Fig. 2:**
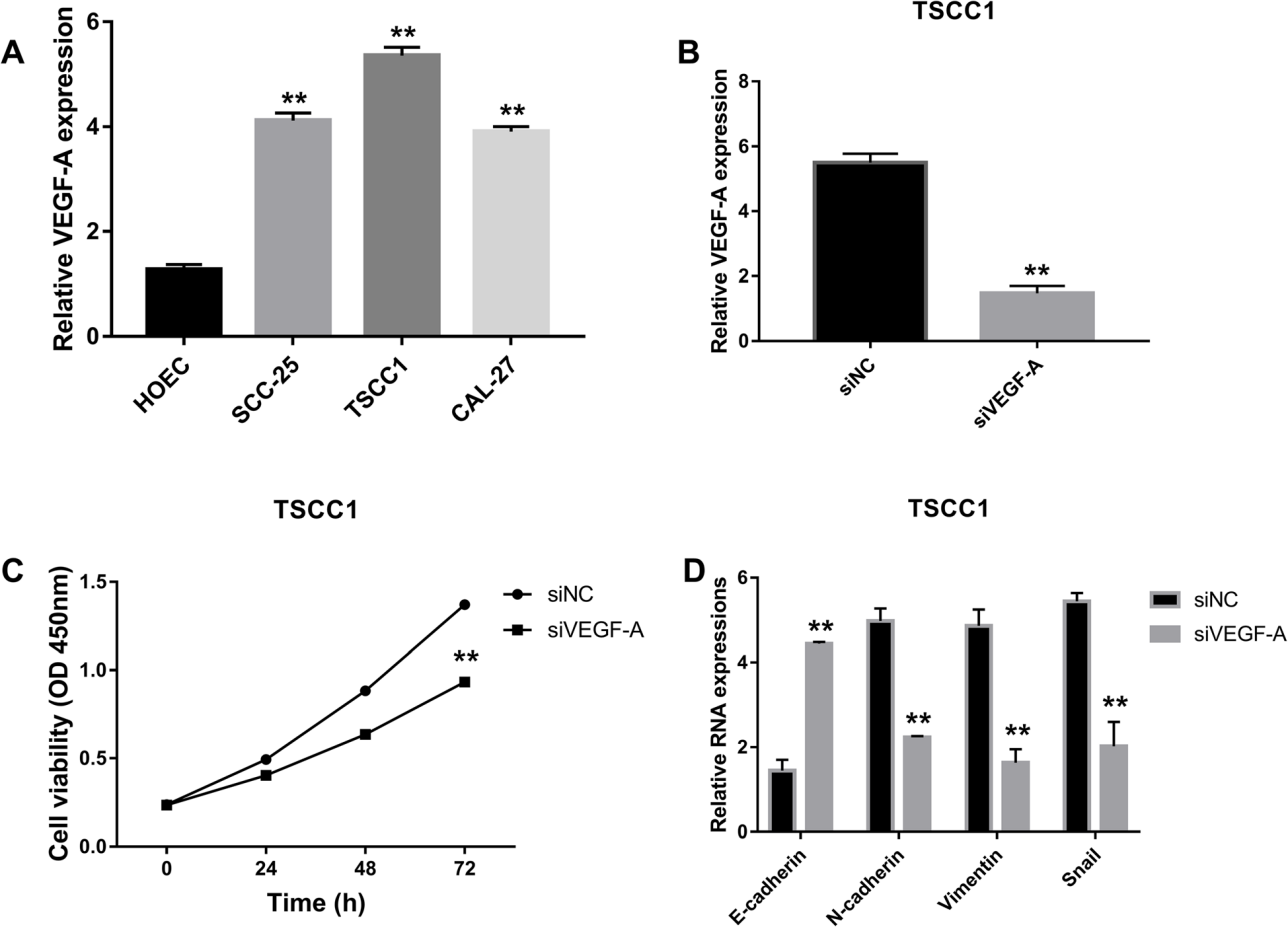
VEGF-A expressed higher in OSCC cell lines with accelerating proliferation and EMT. **a** RNA expressions of VEGF-A were measured in HOEC, SCC-25, TSCC1, and CAL-27 cell lines using RT-qPCR with normalizing to GAPDH and the statistical test was comparing each of the OSCC cell lines to the HOEC cell line, **P < 0.05. **b** RT-qPCR was assessed to evaluated level of VEGF-A after inhibition normalized with GAPDH and the statistical test was comparing siVEGF-A to siNC, **P < 0.05. **c** Cell viabilities were checked by CCK-8 in TSCC1 cell line with suppressed VEGF-A with the statistical test comparing siVEGF-A to siNC, **P < 0.05. **d** RT-qPCR was performed to check RNA levels of E-cadherin, N-cadherin, Vimentin, and Snail with normalizing to GAPDH and the statistical test was comparing E-cadherin, N-cadherin, Vimentin, and Snail in siVEGF-A group to E-cadherin, N-cadherin, Vimentin, and Snail in siNC group, **P < 0.05. Each experiment was repeated three times

After lncRNA NEAT1 was evaluated, VEGF-A was checked. VEGF-A expressions was significantly higher in OSCC cell lines especially in TSCC1 cell line in comparison to HOEC cell line (Fig. 2a). After VEGF-A was suppressed, levels of VEGF-A were checked in TSCC1 cell line, indicating that siVEGF-A obviously resulted in a lower level of VEGF-A in TSCC1 cells line compared with siNC (Fig. 2b). With detecting cell viabilities, siVEGF-A also resulted in much lower viabilities of TSCC1 cell line than siNC (Fig. 2c). In the meantime, results of EMT analysis showed that siVEGF-A could significantly increase expression of E-cadherin while inhibiting levels of N-cadherin, Vimentin, and Snail (Fig. 2d).

### VEGF-A was activating transcription factor of lncRNA NEAT1

**Fig. 3:**
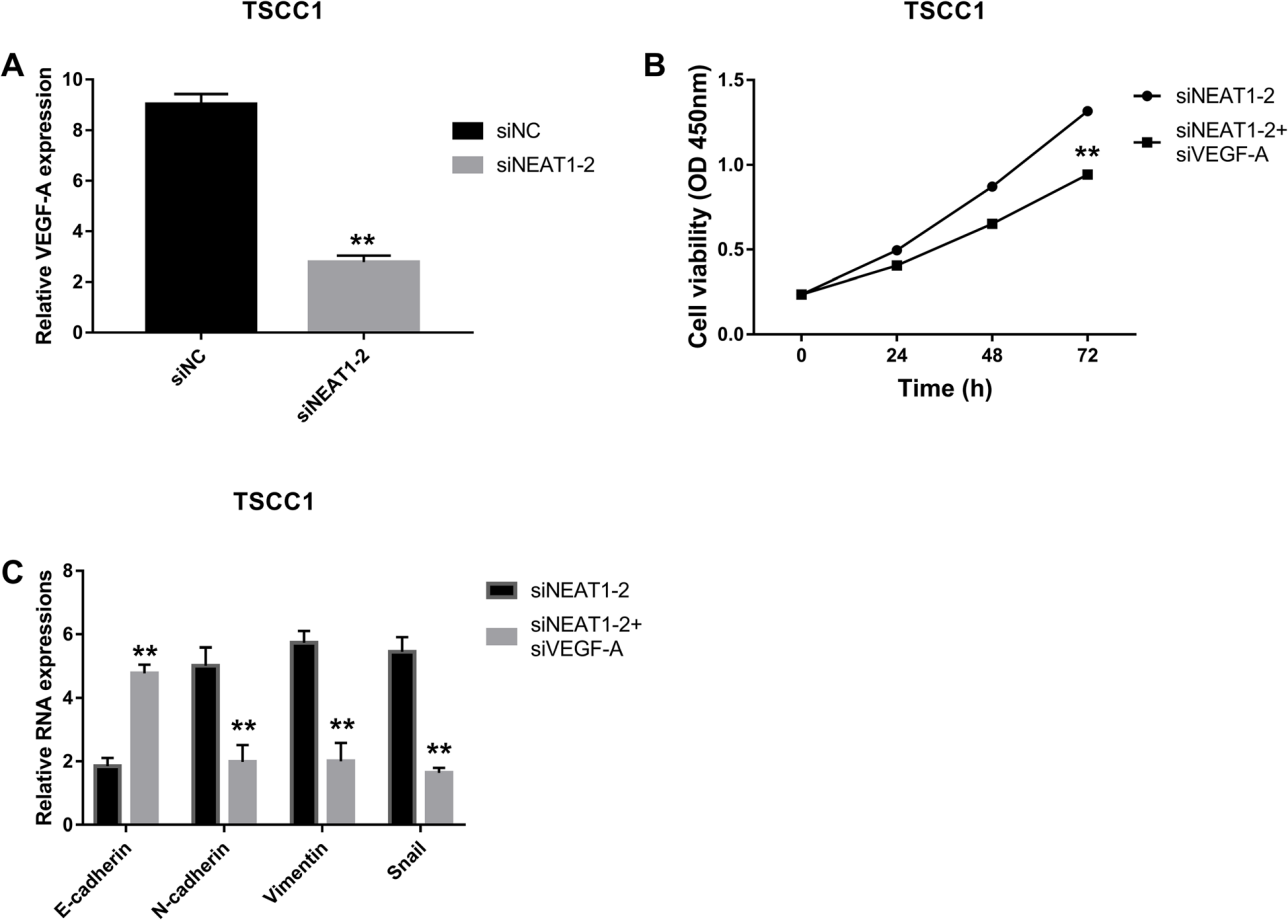
VEGF-A was activator of LncRNA NEAT1 with speeding up proliferation and EMT. **a** Expression of VEGF with inhibited NEAT1-2 were detected by RT-qPCR with normalizing to GAPDH and the statistical test was comparing siNEAT1-2 to siNC, **P < 0.05. **b** CCK-8 was carried out to measure cell viabilities with suppressed NEAT1-2 and VEGF with the statistical test was comparing siNEAT1-2+siVEGF-A to siNEAT1-2, **P < 0.05. **c** E-cadherin, N-cadherin, Vimentin, and Snail expressions with inhibited NEAT1-2 and VEGF-A were measured with RT-qPCR with normalizing to GAPDH and the statistical test was comparing E-cadherin, N-cadherin, Vimentin, and Snail in siNEAT1-2+siVEGF-A group to E-cadherin, N-cadherin, Vimentin, and Snail in siNEAT1-2 group, **P < 0.05. Each experiment was repeated three times

After lncRNA NEAT1 and VEGF-A were examined individually, their correlation was analyzed. After NEAT1 was inhibited, VEGF-A expressions were significantly decreased compared to siNC (Fig. 3a). Then, in detection of cell viabilities, siVEGF-A with SiNEAT1-2resulted in much lower viability of TSCC1 cell line compared with siNEAT1-2 only (Fig. 3b). Besides that, results of EMT analysis showed that siNEAT1-2 with siVEGF-A could apparently enhanced expressions of E-cadherin and reduced levels of N-cadherin, Vimentin, and Snail in the TSCC1 cell line (Fig. 3c).

### LncRNA NEAT1 accelerated proliferation and EMT and repressed apoptosis of OSCC cells via activating Notch signaling pathway

**Fig. 4:**
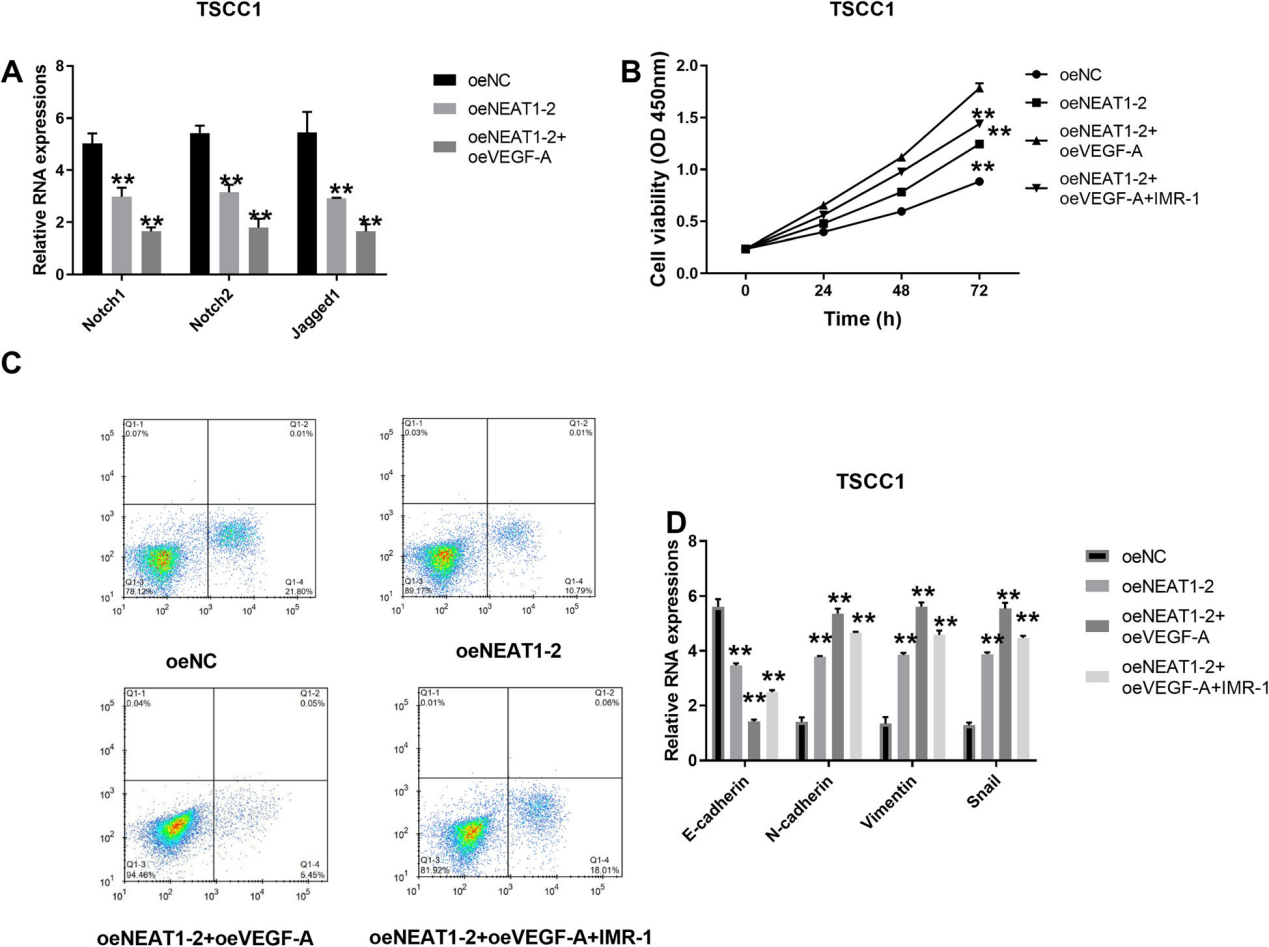
LncRNA NEAT1 accelerated proliferation and EMT and repressed apoptosis of OSCC cells through activating Notch signaling pathway. **a** Expressions of Notch1, Notch2, and Jagged1 with overexpressed NEAT1-2 and VEGF-A were evaluated by RT-qPCR normalized with GAPDH and the statistical test was comparing oeNEAT1-2 and oeNEAT1-2+oeVEGF-A to oeNC, **P < 0.05. **b** Cell viabilities were checked in TSCC1 cells with overexpressed NEAT-1, overexpressed VEGF-A and IMR-1 via CCK-8 with the statistical test was comparing oeNEAT1-2, oeNEAT1-2+oeVEGF-A, and oeNEAT1-2+oeVEGF-A+IMR-1 to oeNC, **P < 0.05. **c** Flow cytometry was performed to measure apoptosis rate of TSCC1 cell line with overexpressed NEAT1-2 and VEGF-A and added IMR-1 with the statistical test was comparing oeNEAT1-2, oeNEAT1-2+oeVEGF-A, and oeNEAT1-2+oeVEGF-A+IMR-1 to oeNC, **P < 0.05. **d** RNA levels of E-cadherin, N-cadherin, Vimentin, and Snail were detected with overexpressed NEAT1-2, overexpressed VEGF-A and IMR-1 using RT-qPCR with normalizing to GAPDH and the statistical test was comparing E-cadherin, N-cadherin, Vimentin, and Snail in oeNEAT1-2, oeNEAT1-2+oeVEGF-A, and oeNEAT1-2+oeVEGF-A+IMR-1 group to E-cadherin, N-cadherin, Vimentin, and Snail in oeNC group, **P < 0.05. Each experiment was repeated three times

Based on previous research, the Notch signaling pathway plays an important role in progression of OSCC. Therefore, whether LncRNA NEAT1 had connection with elements in Notch signaling pathway was analyzed. Compared to the control, oeNC group, Notch1, Notch2, and Jagged1 expressions were all obviously increased in oeNEAT1-2 and oeNEAT1-2 with oeVEGF-A groups and the latter was associated with the highest level of those three factors (Fig. 4a). As the Notch signaling pathway had connections with lncRNA NEAT1 and VEGF-A, IMR-1, a Notch signaling pathway inhibitor, was used to confirm functions of the Notch signaling pathway in the progression of OSCC. Afterwards, CCK-8 was performed to check cell viabilities. This analysis revealed that oeNEAT1-2 and oeVEGF-A groups were associated with higher levels of cell viabilities than oeNEAT1-2 and oeNC groups and adding IMR-1 could retard the promotion of cell viabilities (Fig. 4b). Meanwhile, IMR-1 could heighten apoptosis rate of cells in TSCC1 cell line with oeNEAT1-2 and oeVEGF-A (Fig. 4c). Furthermore, IMR-1 caused a higher expression of E-cadherin and lower levels of N-cadherin, Vimentin, and Snail after compounded with oeVEGF-A and oelncRNA NEAT1-2 (Fig. 4d).

## Discussion

Nuclear enriched abundant transcript 1 (NEAT1) is an important regulator of cell functions and can adjust growth and metastasis of tumors in osteosarcoma [29]. In nasopharyngeal carcinoma, highly expressed lncRNA NEAT1 could accelerate tumor growth through suppressing mIR-124 via NF-κB signaling pathway [30]. A study by Huang et al. revealed that expressions of lncRNA NEAT1 was significantly increased in OSCC cells and knockdown of NEAT1 could repress proliferation of OSCC cells [19]. Therefore, LncRNA NEAT1 was selected in this study for further research in OSCC from a new perspective. First, we examined expressions of LncRNA NEAT1 in normal oral cell line HOEC and OSCC cell lines, SCC-25, TSCC1, and CAL-27. This analysis revealed that LncRNA NEAT1 was highly expressed in OSCC cell lines, especially in the TSCC1 cell line compared to the normal cell line. Therefore, we selected the TSCC1 cell line for the following studies. To knock down the expression of NEAT1 in TSCC1 cell line, we transfected siNEAT1-1 and siNEAT1-2 in to the cells with siNC as a negative control. Our results from RT-qPCR showed that siNEAT1-2 induced a much lower level of lncRNA NEAT1 than siNEAT1-1. Therefore, siNEAT1-2 was chosen for the following experiments. A notable decrease in cell viability in siNEAT1-2 was witnessed compared to siNC group, indicating that suppression of lncRNA NEAT1-2 could block cell viabilities of TSCC1 cells. Moreover, the apoptosis rate was significantly increased with knockdown of lncRNA NEAT1, suggesting that suppression of lncRNA NEAT1 could retard proliferation and induce apoptosis of OSCC cells.

EMT takes part in migration and invasion of cancer cells. Loss of E-cadherin and acquisition of N-cadherin, Vimentin, and Snail can be deemed as signs of EMT, which can be induced in squamous epithelial cells to enhance invasion and migration to accelerate development of OSCC [31–33]. According to research of Takkunen et al., development of EMT in OSCC had connection with downregulation of E-cadherin [34]. In this study, E-cadherin was upregulated while N-cadherin, Vimentin, and Snail were all inactivated in siNEAT1-2 group, indicating the deterrence of EMT process was correlated with the inhibition of NEAT1. Therefore, LncRNA NEAT1 could regulate EMT to expedite the progression of OSCC in vitro.

Tumor angiogenesis plays an important role in the oncogenesis of in malignant tumors. Vascular endothelial growth factor (VEGF) has been acknowledged as the most important tumor angiogenesis factor [35]. In the VEGF family, VEGF-A has been shown to promote vascular endothelial growth [35], playing an important role in disease related to angiogenesis, especially in cancers [36]. VEGF-A expression was over 50 times higher in serum and in immunohistochemical tests in OSCC compared to healthy control groups and VEGF-A expression had positive association with malignant lesions in oral cavity [37]. Besides that, Gordana Supic et al. revealed that VEGF-A was significantly correlated with lower overall survival rate of patients with OSCC [38]. Therefore, in this study, we also observed VEGF-A changes in response to NEAT1 or NOTCH signaling changes as another indicator of OSCC development. Same as lncRNA NEAT1, VEGF-A was significantly increased in OSCC cell lines compared to HOEC cell line. Meanwhile, TSCC1 cell line had the highest level of VEGF-A. After knocking down VEGF-A in TSCC1 cells, expression of VEGF-A declined and cell viabilities were downregulated in comparison to the control group. As for EMT, siVEGF-A induced E-cadherin and inhibited N-cadherin, Vimentin, and Snail expressions significantly. Therefore, siVEGF-A could downregulate proliferation and EMT, indicating that it could be a regulatory factor in development in OSCC. Moreover, correlation between lncRNA NEAT1 and VEGF-A were analyzed and results showed that expression of VEGF-A could be reduced by siNEAT1-2 in TSCC cells. Furthermore, the combination of siNEAT1-2 and siVEGF-A resulted in lower cell viability in TSCC1 cell line than that in siNEAT1 group. As for EMT, both siNEAT1-2 and siVEGF-A could increase E-cadherin and inhibit N-cadherin, Vimentin, and Snail in TSCC1 cell line. These results suggest that VEGF-A expression could be regulated by lncRNA NEAT1 and inhibition of VEGF-A could impede the function of lncRNA NEAT1 in the proliferation and EMT of OSCC.

Notch was first discovered in 1917 and was named Notch because of the mutation causing deformities of fly’s wings. The Notch signaling pathway is a chained signaling pathway constituted by ligands, receptors, and down-stream DNA binding proteins. Jagged1 is a ligand and Notch1 and Notch2 are two kinds of receptors. Moreover, functions of Notch signaling pathway in OSCC have also been validated. Thanaphum Osathanon et al. have explored roles of Notch signaling pathway in OSCC, demonstrating that suppression of Notch signaling pathway significantly decreased proliferation of OSCC cells in vitro [39]. Furthermore, Ryoji Yoshida et al. have studied Notch1 with OSCC, proving that Notch1 could accelerate the development of OSCC and that inactivation of Notch could be a helpful way to treat patients suffering from OSCC [40]. Notch signaling pathway was investigated in this study in correlation with NEAT1 and VEGF-A. Our results pointed out that Notch1, Notch2, and Jagged1 expressions increased with overexpression of NEAT1-2 and VEGF-A. In order to understand how the Notch signaling pathway interacts with lncRNA NEAT1 and VEGF-A, IMR-1, a Notch signaling pathway inhibitor, Inhibitor of Mastermind Recruitment-1 (IMR-1) was applied. Cell viabilities were diminished and apoptosis rate was significantly promoted by IMR-1, compared to cells transfected with overexpressed NEAT1 and VEGF-A. Moreover, IMR-1 induced E-cadherin and inhibited N-cadherin, Vimentin, and Snail, curbing EMT process, suggesting that inhibition of the Notch signaling pathway could partially counteract functions of lncRNA NEAT1 and VEGF-A, consequently inhibiting cell proliferation and EMT process while enhancing apoptosis in OSCC, which is in consistence with the previous finding that IMR-1 could inhibit the growth of patient-derived tumor xenografts [24].

## Conclusion

LncRNA NEAT1 could activate VEGF-A to accelerate progression of OSCC via Notch signaling pathway. Furthermore, Notch inhibitor IMR-1 exerts inhibitory impact on OSCC cells through interaction with NEAT1 and VEGF-A. However, we still need further research in vivo and in clinical stages to gain a further understanding of the interplays among NEAT1, VEGF-A, and Notch signaling in OSCC.

## Data Availability

The datasets used and/or analyzed during the current study are available from the corresponding author.

## Abbreviations

OSCC: Oral squamous cell carcinoma
NEAT1: Nuclear paraspeckle assembly transcript 1
ATCC: American Type Culture Collection
FBS: Fetal bovine serum
VEGF: Vascular endothelial growth factor
EMT: Epithelial-to-mesenchymal transition
