# Correction to: LncRNA NEAT1 mediates progression of oral squamous cell carcinoma via VEGF-A and Notch signaling pathway

**DOI:** 10.1186/s12957-020-02050-z

**Published:** 2020-10-27

**Authors:** Ke He, Zhi-Bin Zhu, Rui Shu, Ai Hong

**Affiliations:** 1 Department of Stomatology, Chengdu Seventh People’s Hospital, Chengdu, 610015 Sichuan China; 2 Department of Orthodontics and Pediatric Dentistry, West China School of Stomatology State Key Laboratory of Oral Diseases, Sichuan University, Chengdu, 610041 China; 3 Department of Stomatology, The Third Affiliated Hospital of Sun Yat-sen University, Guangzhou, 510000 China

**Correction to: World J Surg Onc 18, 261 (2020)**

**https://doi.org/10.1186/s12957-020-02028-x**

Following publication of the original article [1], the authors identified an error in the first author name.

The incorrect author name is: He Ke

The correct author name is: Ke He

The author group has been updated above and the original article [1] has been corrected.
